# A Rare Colonization in Peritoneum After Blunt Abdominal Trauma: *S. putrefaciens* and *S. cerevisiae*

**DOI:** 10.4274/balkanmedj.2017.0773

**Published:** 2018-07-24

**Authors:** Oana Bulat, Cristian Bulat, Mihaela Blaj, Ioana Lupusoru, Viorel Scripcariu

**Affiliations:** 1 Department of Surgery, “Sf. Spiridon” Hospital, “Grigore T. Popa” University School of Medicine and Pharmacy, Iasi, Romania; 2 Department of Intensive Care Unit, “Sf. Spiridon” Hospital, “Grigore T. Popa” University School of Medicine and Pharmacy, Iasi, Romania; 3 Department of Oncology Surgery, Regional Institute of Oncology, “Grigore T. Popa” University School of Medicine and Pharmacy, Iasi, Romania

**Keywords:** Blunt trauma, gram-negative bacteria, peritonitis, S. cerevisiae, S. putrefaciens

## Abstract

**Background:**

*Shewanella* spp. are gram-negative bacteria, saprophytes, and rarely pathogenic. *Saccharomyces cerevisiae* is the well-known yeast used for fermentation in industry and molecular biology for research. In humans, it is a very rare pathogen which colonizes the digestive tract, and its utility has been linked to the treatment and prevention of diarrhea associated with *Clostridium difficile*.

**Case Report:**

A 27-year-old male, victim of aggressive, blunt trauma with a 4-day history of symptoms was admitted to our surgery unit. Abdominal sonography revealed peritoneal fluid in all spaces with fibrin. We performed laparotomy and observed perforations on the ileum and general peritonitis with pus. Following surgery, patient was admitted to the intensive care unit with septic shock. The antibiogram from the peritoneal liquid revealed *S. putrefaciens* and *S. cerevisiae*.

**Conclusion:**

Although very rare, *S. putrefaciens* and *S. cerevisiae* may colonize in the peritoneum after blunt abdominal trauma.

*Shewanella* spp. are gram-negative bacteria, saprophytes, and rarely pathogenic (1). Initially isolated in 1931, they belong to the marine environment; *Shewanella* (*Pseudomonas*) *putrefaciens* is found in water, soil, and animal sources (1) and produces hydrogen sulfide.

*Saccharomyces cerevisiae* is the well-known yeast used for fermentation in industry and molecular biology for research. In humans, it is a very rare pathogen which colonizes the digestive tract, and its utility has been linked to the treatment and prevention of diarrhea associated with *Clostridium difficile* (2).

We report a case of delayed peritonitis due to blunt abdominal trauma, with septic shock and a positive antibiogram for both *Shewanella putrefaciens* and *S. cerevisiae* from the intraperitoneal liquid.

## CASE PRESENTATION

**Figure 1:**
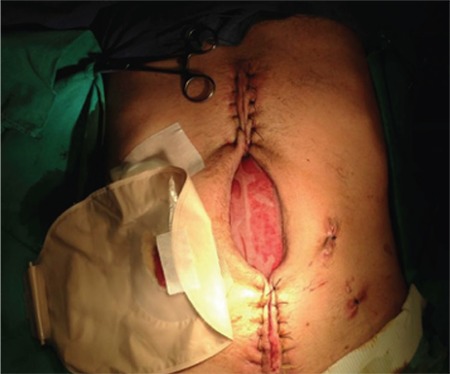
Progressive closure of the abdomen.

A 27-year-old male, victim of aggressive, blunt trauma with a 4-day history of symptoms was admitted to our surgery unit. The patient presented abdominal pain, was hypotensive (100/50 mmHg), perspiring, and drowsy. The abdominal examination revealed diffuse tenderness and rebound tenderness to palpation, 2 periumbilical ecchymoses of 2/2 cm, many others within the left deltoid area and on the lower limbs. Abdominal sonography revealed peritoneal fluid in all spaces with fibrin, chest X-rays unremarkable. Laboratory investigation showed hemoglobin and white blood cell count with normal values, creatinine 4.04 mg/dL, urea 209 mg/dL, mild acidosis, lactate 21 mmol/L, creatinine-kinase 1910 U/L, lactate-dehydrogenase 451 U/L, and ethanol 34 mg/dL. During laparotomy, we discovered 2 perforations on the ileum and general peritonitis with pus. We performed an enterectomy, about 20 cm of ileum, and an end-ileostomy with a mucous fistula on the distal ileum, saline lavage, four drainages, and a laparostomy. Following surgery, the patient was admitted to the intensive care unit (ICU) with septic shock and multiple organ dysfunction syndrome (MODS). We initiated empirical therapy with broad-spectrum antibiotics with ertapenem and vancomycin. The antibiogram from the peritoneal liquid revealed *S. putrefaciens*, *S. cerevisiae, *and* Candida *species susceptible to amikacin, gentamicin, ciprofloxacin, third-generation cephalosporins, and fluconazole. On the second day following surgery, we intervened again, and we discovered multiple intraperitoneal abscesses. We washed the patient once more, drained, and treated him surgically with a laparostomy. From the peritoneal liquid, *Acinetobacter baumannii *developed that were susceptible to colistin, and the same bacteria was discovered within the tracheal secretion. The patient remained on mechanical ventilation, inotropic support, and sedated. Fever (38.5 ºC), leukocytosis (22850/μL), high lactate (31.5), very high inflammatory markers (presepsin, procalcitonin, C-reactive protein), and higher inotropic support (from 10 to 25 μg/hour of norepinephrine) convinced us to repeat the intervention including both washing and laparostomy with negative pressure. Until the 11^th^ day following surgery, the patient remained intubated**, **with inotropic support, had high fever (38 ºC), and very grave general status-MODS. He also had septic shock, with a positive antibiogram from the venous catheter for *Klebsiella pneumoniae* susceptible to sulfamethoxazole with trimethoprim, and for *Staphylococcus hominis* susceptible to vancomycin and levofloxacin. From the tracheal secretion, the bacteria found in the peritoneal liquid (*A. baumannii*) was sampled analogously. Every other day, we repeated the intervention to change the kit for intra-abdominal negative pressure. On the 12^th^ day following surgery, the general status of the patient had improved. Thus, he no longer remained intubated, was hemodynamically stable, without inotropic support, with spontaneous respirations, and had a functional ileostomy. We changed 6 kits of negative abdominal pressure, the antibiogram from the peritoneal cavity was positive once again for *Klebsiella* and *Acinetobacter* with antibiotic therapy (Figure 1). During the last 3 interventions, we progressively closed the abdomen, and upon the 10^th^ repeated intervention, we closed the entire abdomen, after which the patient was discharged from the ICU after 20 days where he had been administered many doses of antibiotherapy. The patient was discharged from the surgery unit on the 30^th^ day after admission and had completely recovered.

**Figure 2:**
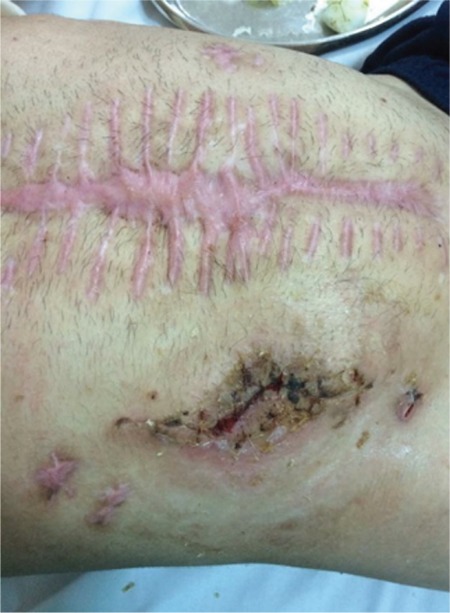
Median postoperative hernia and closed ileostomy.

**Figure 3:**
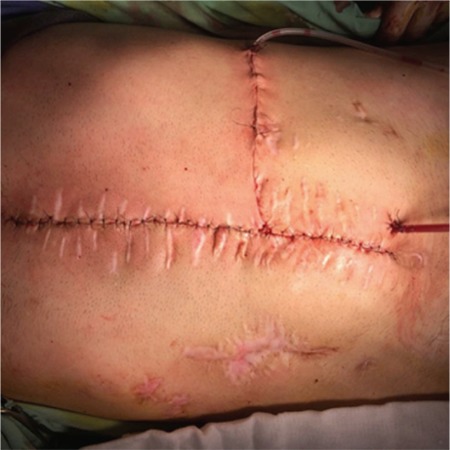
Musculo-cutaneous flap.

After 6 months, the patient returned to close the ileostomy, and after another 8 months, he returned with a median postoperative hernia (Figure 2). Given both the large defect and due to the weight gained, we chose to resolve the hernia with an intraperitoneal mesh. This method was unsuccessful, and even the antibiogram was negative; the patient developed ulceration of the skin which was resolved surgically. We removed the mesh and performed a muscular-cutaneous flap (Figure 3). Written informed consent was obtained from the patient who participated in this case.

## DISCUSSION

Although* Shewanella *spp. infections have been reported worldwide, and publications were single-case reports or a small series. Initially known as *Achromobacter putrefaciens*, in 1941 the bacteria were subsequently reclassified as *P. putrefaciens.* It was not until its reclassification into the family *Vibrionaceae* and named after James Shewan in honor of his work in marine microbiology (3), 30 species and infections have been identified. These infections are usually associated with underlying conditions (low socio-economic status, poor personal hygiene, immunocompromized hosts with a history of contact with seawater or ingestion of raw seafood), health complications, and very often isolated in polymicrobial infections. The most important risk factors for human infection with *Shewanella* are immunocompromized patients with malignancies, severe heart failure, renal failure,hepato-biliary disease, neutropenia, and chronic ulcerations on the lower extremities (4). *Shewanella* spp. is usually susceptible to common antibiotics, including aminoglycosides, erythromycin, quinolones, and carbapenems. Susceptibility to cephalosporins is variable, with more isolates being susceptible to both third- and fourth-generation compared with first- and second-generation cephalosporins and are resistant to penicillin (1).

In our case, the patient had no history of exposure to seawater or ingestion of raw seafood, and he was not immune-compromised. The antibiogram with *Shewanella* susceptible to third generation cephalosporins shows that the bacteria were mostly in the gastrointestinal tract.

*S. cerevisiae* is usually non-pathogenic, but we can find in literature a series of infection cases at the immune-deficient, injured or debilitated patients (5). Uncommon yeast infections, such as *Saccharomyces*, although rare, are increasing, being isolated in up to 4% of blood cultures (6). In our case, it was interpreted as “possible contamination,” but in literature it has been cited the relationship between *Shewanella* and *Saccharomyces*. Both of these organisms have an enzyme, fumarate reductase (FRD), which reduces fumarate to succinate (7). There are two types of FRD (8): the typical group is membrane-bound, the other is a soluble monomer. Few organisms such as the *Shewanella *species, *S. cerevisiae*, and *Trypanosoma brucei* (7) possess the latter type of FRD. Thus, the association between these two microorganisms in our case (*Shewanella* and *Saccharomyces*) is not fortuitous. Furthermore, the literature describes the association between *Saccharomyces* and *Candida* as the cause of high mortality rates but mentions that early fungal therapy improves outcomes (9). Hence, the association between ciprofloxacin and fluconazole from the beginning was successful for our patient.

Two others pathogens isolated during the ICU stay are multi-drug-resistant bacteria that are members of the six bacteria responsible for nosocomial infections, better known under the acronym “ESKAPE” (*E. faecium*, *S. aureus*, *K.* *pneumoniae*, *A. baumannii*, *P. aeruginosa*, and *Enterobacter* spp.) (10).

In conclusion, although very rare, *S. putrefaciens* and *S. cerevisiae* may colonize in the peritoneum after blunt abdominal trauma.
